# Complete Genome Sequences of Four Serotypes of Dengue Virus Prototype Continuously Maintained in the Laboratory

**DOI:** 10.1128/MRA.00199-19

**Published:** 2019-05-09

**Authors:** Kwanrutai Chin-inmanu, Aroonroong Suttitheptumrong, Duangjai Sangsrakru, Dumrong Mairiang, Sithichoke Tangphatsornruang, Prida Malasit, Prapat Suriyaphol

**Affiliations:** a Division of Bioinformatics and Data Management for Research, Research Group and Research Network Division, Faculty of Medicine Siriraj Hospital, Mahidol University, Bangkok, Thailand; b Center of Excellence in Bioinformatics and Clinical Data Management, Research Group and Research Network Division, Faculty of Medicine Siriraj Hospital, Mahidol University, Bangkok, Thailand; c Division of Molecular Medicine, Research Department, Faculty of Medicine Siriraj Hospital, Mahidol University, Bangkok, Thailand; d National Center for Genetic Engineering and Biotechnology (BIOTEC), National Science and Technology Development Agency (NSTDA), Pathum Thani, Thailand; e Division of Dengue Hemorrhagic Fever Research, Research Department, Faculty of Medicine Siriraj Hospital, Mahidol University, Bangkok, Thailand; f Molecular Biology of Dengue and Flaviviruses Research Team, National Center for Genetic Engineering and Biotechnology (BIOTEC), National Science and Technology Development Agency (NSTDA), Pathum Thani, Thailand; DOE Joint Genome Institute

## Abstract

Dengue prototype strains are widely used for virological study. The strains presented here have been cultured under different laboratory environments, resulting in accumulating genetic variations. We present the genomes of four serotypes of the dengue prototype strain that were continuously maintained in the laboratory. These genomes contain bases different from those of the original prototype strains in GenBank.

## Abstract (precis)

Dengue prototype strains are widely used for virological study. The strains presented here have been cultured under different laboratory environments, resulting in accumulating genetic variations.

## ANNOUNCEMENT

Dengue virus (DENV) is the most prevalent arbovirus and is found mainly in tropical and subtropical regions. The virus belongs to the family *Flaviviridae* and the genus *Flavivirus*. Four serotypes of dengue virus are a cause of dengue fever (DF), dengue hemorrhagic fever (DHF), and dengue shock syndrome (DSS). The prototype strains of four dengue serotypes frequently used in the laboratory are DENV1 strain Hawaii, DENV2 strain 16681 and strain New Guinea C (NGC), DENV3 strain H87, and DENV4 strain H241. The prototype strains have been cultured and used for dengue study in laboratories since 1980 (1). A few complete genome records of dengue prototype strains have been submitted to the GenBank database, as described in Table 1. Since dengue virus has a high mutation rate during genome replication (2), some variations may be accumulated during continuous propagation in the laboratory. We sequenced five prototype strains of dengue virus which have been propagated and used for studying dengue virus in our laboratory.

**TABLE 1:** Details of genome assemblies and their base changes compared with previously submitted reference genomes

Serotype and strain	No. of reads	Genome coverage (×)	Position on genome covered	Comparison to previously submitted reference genomes	
				Reference GenBank accession no. (release/modification date) (reference no.)	No. of base changes/substitutions
DENV1 Hawaii	18,015	385	1–10701	KM204119 (2016) (9)	36
DENV2 16681	33,138	699	1–10688	M84727 (1993) (10)	67
				U87411 (1997) (11)	9
				KU725663 (2016) (12)	10
DENV2 NGC	6,546	139	1–10688	M29095 (1993) (13)	14
				AF038403 (1998) (14)	11/1 (delete)^*a*^
				KM204118 (2016) (9)	6
DENV3 H87	22,947	485	12–10661	M93130 (1996) (15)	36
				KU050695 (2016) (9)	7
DENV4 H241	26,958	578	1–10621	AY947539 (2005)^*b*^	13/1 (insert)^*a*^
				KR011349 (2016) (9)	16

a Deletions and insertions were found in the untranslated region (UTR).

b Direct submission by the Dengue Unit, Novartis Institute for Tropical Diseases, Singapore.

The dengue viruses presented here were from prototype strains of the four serotypes, namely, DENV1 strain Hawaii, DENV2 strain 16681 and strain NGC, DENV3 strain H87, and DENV4 strain H241. All dengue viruses were propagated in C6/36 Aedes albopictus cells by the cell culture laboratory of the Division of Dengue Hemorrhagic Fever Research (Faculty of Medicine Siriraj Hospital, Mahidol University, Thailand). Five prototype strains were maintained in the laboratory for more than 10 years. The prototype strains were prepared for sequencing by using the 454 pyrosequencing GS FLX platform (Roche Applied Science) in the same run as that reported in our previous study (3). The total read count of the pooled sequencing results was 134,441 reads, and the average read length was 232 bp. Each read was then aligned to reference genomes (GenBank accession numbers KM204119, U87411, M29095, M93130, and AY947539) using BLASTn version 2.7.1+ (4) with default parameters and classified to the best-matched prototype strains. The number of reads that mapped to each reference genome is shown in Table 1. The depths of genome coverage of all five strains were more than 100× and covered from the first base on the genome except for that of DENV3 strain H87, which started from the 12th base (Table 1). The consensus genome sequences were assembled using VICUNA version 1.3 (5). To identify the false insertions/deletions (indels) introduced in the assembly process, classified reads of each strain were aligned to the corresponding assembled genome using BWA-MEM version 0.7.17 (6). False indels were revealed by exploring the alignment result in the Integrative Genomics Viewer (IGV) tool, version 2.4.14 (7, 8) and removed from assembled genome sequences. Variations of our genome sequences compared with those of prototype strain sequences deposited in GenBank are shown in Table 1.

### Data availability.

The high-throughput sequencing data of five prototype strains have been deposited in the Sequence Read Archive (SRA) database under the accession number SRR7428480. The complete genome sequences were deposited in the GenBank database under the accession numbers MK506262, MK506263, MK506264, MK506265, and MK506266.
